# Hybrid Methods for Fundus Image Analysis for Diagnosis of Diabetic Retinopathy Development Stages Based on Fusion Features

**DOI:** 10.3390/diagnostics13172783

**Published:** 2023-08-28

**Authors:** Mohammed Alshahrani, Mohammed Al-Jabbar, Ebrahim Mohammed Senan, Ibrahim Abdulrab Ahmed, Jamil Abdulhamid Mohammed Saif

**Affiliations:** 1 Computer Department, Applied College, Najran University, Najran 66462, Saudi Arabia; iaalqubati@nu.edu.sa; 2 Department of Artificial Intelligence, Faculty of Computer Science and Information Technology, Alrazi University, Sana’a, Yemen; 3 Computer and Information Systems Department, Applied College, University of Bisha, Bisha 67714, Saudi Arabia; jasaif@ub.edu.sa

**Keywords:** CNN, FFNN, hybrid models, hybrid features, diabetic retinopathy, handcrafted

## Abstract

Diabetic retinopathy (DR) is a complication of diabetes that damages the delicate blood vessels of the retina and leads to blindness. Ophthalmologists rely on diagnosing the retina by imaging the fundus. The process takes a long time and needs skilled doctors to diagnose and determine the stage of DR. Therefore, automatic techniques using artificial intelligence play an important role in analyzing fundus images for the detection of the stages of DR development. However, diagnosis using artificial intelligence techniques is a difficult task and passes through many stages, and the extraction of representative features is important in reaching satisfactory results. Convolutional Neural Network (CNN) models play an important and distinct role in extracting features with high accuracy. In this study, fundus images were used for the detection of the developmental stages of DR by two proposed methods, each with two systems. The first proposed method uses GoogLeNet with SVM and ResNet-18 with SVM. The second method uses Feed-Forward Neural Networks (FFNN) based on the hybrid features extracted by first using GoogLeNet, Fuzzy color histogram (FCH), Gray Level Co-occurrence Matrix (GLCM), and Local Binary Pattern (LBP); followed by ResNet-18, FCH, GLCM and LBP. All the proposed methods obtained superior results. The FFNN network with hybrid features of ResNet-18, FCH, GLCM, and LBP obtained 99.7% accuracy, 99.6% precision, 99.6% sensitivity, 100% specificity, and 99.86% AUC.

## 1. Introduction

Diabetic retinopathy, a retina disease generated by diabetes, damages the retina and leads to blindness. In DR, blood glucose rises above its normal level, leading to leakage of some fluids and blood to the retina [1]. Proliferative diabetic retinopathy (PDR) is a more advanced stage of DR. It is characterized by the growth of new blood vessels in the retina. These new blood vessels are fragile and can leak blood or fluid, which can damage the retina and lead to vision loss. Non-proliferative diabetic retinopathy (NPDR) is the earliest stage of DR. It is characterized by small blood vessel changes in the retina. NPDR may not cause any symptoms, but it can lead to more serious complications if it is not treated. NPDR is divided into mild, moderate, and severe.

Mild NPDR: This stage is characterized by the presence of microaneurysms, which are small bulges in the blood vessels of the retina. Microaneurysms may not cause any symptoms, but they can be a sign of early damage to the retina. Circular red dot marks appear at the end of the micro-aneurysm (MA) [2].

Moderate NPDR: This stage is characterized by the presence of microaneurysms, as well as other changes in the blood vessels of the retina, such as hemorrhages and exudates. Hemorrhages are small spots of blood that leak from the blood vessels, while exudates are small areas of fluid that leak from the blood vessels. Moderate NPDR can cause some vision loss, such as blurred vision or dark spots in the field of vision. In the moderate stage, red dots in the MA expand into deeper layers, and a flame hemorrhage occurs in the retina.

Severe NPDR: This stage is characterized by the presence of many microaneurysms, hemorrhages, and exudates. Severe NPDR can cause significant vision loss, such as tunnel vision or complete blindness. Neovascularization occurs and grows on the retina’s inner surface of the retina [3].

**Figure 1:**
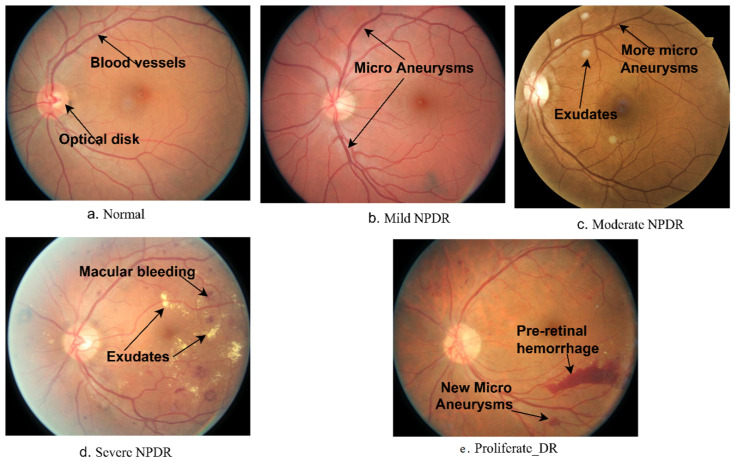
Stages of DR development with the appearance of biomarkers.

Figure 1 shows the stages of DR development with biomarkers appearing at each stage. According to the World Health Organization (WHO) reports, more than 422 million people were diagnosed with diabetes in 2014, and more than a third of them (35%) were infected with DR due to damage to the delicate blood vessels of the retina [4]. The number of people with DR will increase considerably to 592 million by 2025 [5]. The prevalence of DR also differs between groups of diabetics according to their classification, as estimates in the United States indicate that 86% of people with type 1 diabetes and 40% with type II suffer from DR [6]. Vision loss varies from one person with diabetes to another according to the development stages of DR. About 10% of people with diabetes who do not have DR will develop any stage of NPDR, and patients with severe NPDR have a PDR risk of 75%. Therefore, all stages of DR are a measure of the risk of developing international clinical DR disease [7].

Treatment options vary among different stages of DR. Diabetics with DR or stage 1 NPDR (mild) need only regular check-ups. Patients with moderate or severe NPDR require treatment with scatter laser or vitrectomy. Therefore, determining the stage of DR is important to provide appropriate treatment to patients. The diagnosis of NPDR involves fundus imaging and analysis by an ophthalmologist. In the stage of PDR, new abnormal blood vessels will form, which are fragile and burst and bleed, leading to blindness. The most helpful prognosis for effective treatment is in the NPDR stages. Therefore, regular fundus examination for diabetic patients is an effective clinical method for the detection of abnormal blood vessels [8].

However, DR examinations require clinical knowledge, highly qualified and experienced ophthalmologists, and time to analyze fundus images and detect DR in its early stages. The number of diabetic patients worldwide has increased from 4.7% to 8.5% from 1980 to 2014 [9,10]. In this regard, the number of skilled ophthalmologists is rare and unequally distributed; in 2012, the number of doctors globally reached 210,730 (i.e., three doctors for every 100,000 people) [11]. The gap is also very wide between ophthalmologists and diabetics in developing countries. Thus, automated diagnosis using artificial intelligence techniques is necessary for the detection of DR. In literature, many methods have been implemented to detect the evolution of DR by CNN models and machine learning. The main and most difficult task lies in feature extraction methods. Representative features are extracted from fundus images to diagnose DR with maximum accuracy and effectiveness and reduced computational time. In the proposed work, features are extracted by CNN models and classified by a machine learning algorithm. Features were extracted from retinal images using a hybrid approach that combined features extracted by CNN with handcrafted features extracted using features from FCH, GLCM, and LBP. This hybrid approach was able to extract features that represent each stage of DR with high accuracy. The hybrid approach used in this study was able to extract features that represent each stage of DR with high accuracy. This is because the CNN models were able to learn features specific to DR, while the handcrafted features were able to extract features general to images. The combination of these two types of features resulted in a model that was able to classify DR with high accuracy accurately. This method is novel and one of the main contributions of this study. The hybrid approach used in this study is a promising new DR detection and classification method.

The main essential contributions to this work:

- Enhancement of fundus images with average and Laplacian filters and merging the filters’ outputs to obtain an improved image.
- Diagnosing fundus images by using a hybrid technique between CNN models and SVM algorithm.
- Applied the FFNN network based on hybrid features of GoogLeNet, and handcrafted as well as ResNet-18 and handcrafted.

The remainder of the paper is organized as follows. Section 2: This section summarizes several relevant previous studies on DR detection and classification. Section 3: This section describes the methodology and materials used to analyze fundus images and identify the stages of DR development. Section 4: This section summarizes the evaluation results achieved by the proposed system. Section 5: This section compares the proposed system’s performance with other state-of-the-art methods. Section 6: This section concludes the paper and discusses future work.

## 2. Related Work

We reviewed several studies that have used fundus images to detect DR early. Many researchers have used various methods to achieve promising results. Our study distinguishes itself from previous works by extracting hybrid features through CNN models and combining them with handcrafted features. Our methodology is novel and powerful for representing the most critical features of each stage of DR development. It uses a hybrid technique that combines CNN models and an SVM algorithm. Our proposed method yields superior results for the detection of the developmental stages of DR.

Liu et al. proposed three models to improve fundus imaging diagnosis of DR. Basic models such as EfficientNetB4, NASNetLarge, and InceptionResNetV2 were selected and trained by cross-entropy loss enhancement. The outputs of these models were used to train the hybrid models. The models achieved an accuracy between 85.44% and 86.34% [12]. Qummar et al. introduced five CNN models for fundus imaging diagnosis by encoding rich features. The models obtained good results in distinguishing the stages of DR [13]. Gao et al. presented the Inception-V3 model for DR data set diagnostics; They standardized the coordination of all fundus images through a pipeline. They submitted a proposed modification to the model and evaluated its performance with other CNN models. The model achieved high performance in the diagnosis of DR [14]. Gayathri et al. developed a CNN model to extract features and diagnose them by machine learning. The J48 algorithm based on features outperformed the rest of the machine learning [15]. Wan et al. presented CNN models to diagnose a set of fundus images and overcome the challenges of segmentation, classification, and detection of DR. They trained and tested the data set provided by Kaggle and achieved good results in the diagnosis of DR images [16]. Frank et al. introduced an improved hybrid system (IDx-DR-EU-2.1; IDx) for detecting DR before it leads to blindness. The hybrid system operates classification of DR and (IDx-DR-EU-2.1) has achieved superior results compared with the reference standard [17]. Romany et al. presented the AlexNet for the diagnosis of DR. The model includes an image enhancement mechanism, segmentation of an area of interest based on connected component analysis, feature extraction by linear discriminant analysis, and diagnosis by SVM. AlexNet showed better results with FC7 features, where it achieved an accuracy of 97.93%, while with PCA features, it achieved an accuracy of 95.2% [18]. Shanthi et al. developed a CNN model to classify the Messidor data set. Convolutional layers were used to extract features, and the ReLU layer was used to optimize the features. The model yielded good results in distinguishing the stages of DR development [19]. Tao et al. presented a CNN model to diagnose 13,673 images divided into six classes. The system segmented a region of interest to detect DR stages and evaluated a deep learning model on the DDR data set [20]. Martinez et al. presented ResNet50 to extract the most important representative features without image optimization. The ResNet50 model was evaluated on the MESSIDOR data set, which achieved good results for each class in the data set [21]. Hemanth et al. presented a hybrid system of CNN and image processing for DR diagnosis. The hybrid method was evaluated on 400 images from the MESSIDOR data set, which achieved good results for classifying fundus images [22]. Lifeng et al. a CNN model for analysis of fundus image of aneurysm for detection of PDR. The system classified fundus images as normal or abnormal by semantic segmentation and dividing image pixels based on aneurysm features [23]. Chenrui et al. designed a Source-Free Transfer Learning methodology for DR diagnosis through two modules, namely, Collaborative Consistency and Target Generation. The target generation unit trains data, and the collaborative consistency unit improves the methodology through the target unit images. The method was evaluated on the APTOS 2019 data set and achieved good results for diagnosis using fundus images [24].

## 3. Materials and Methods

**Figure 2:**
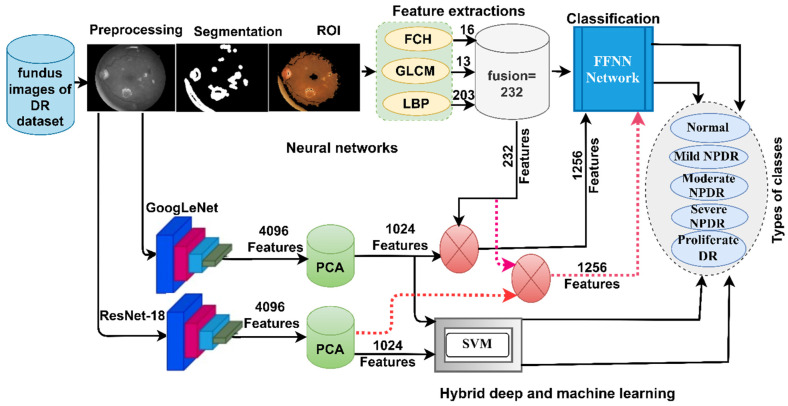
Methodology of color fundus image diagnostics for detection of DR developmental stages.

This section describes the methods and materials used in the study to analyze and diagnose DR stages using fundus images. The study proposes two methods: Diagnosis of fundus images using a hybrid technique that combines CNN models and the SVM algorithm. Analysis of fundus images using the FFNN network based on the hybrid features extracted using the CNN models, followed by dimensionality reduction using the PCA algorithm and combining them with handcrafted features (Figure 2).

### 3.1. Data Set Description

**Figure 3:**
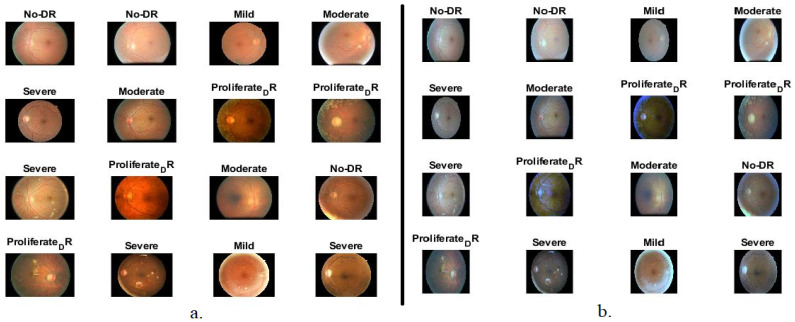
A set of fundus image samples (**a**) before enhancement (set of data set) (**b**) after enhancement.

The data set has been presented to researchers interested in computer-aided diagnosis of DR and is available on Kaggle [25]. The data set consisted of 35,126 fundus images in 24-bit RGB color space with a resolution of 3500 × 3000 pixels. The data set has been classified by many experts and trainers into five classes: 25,810 images of normal at 73.48%, 2443 images of mild NPDR at 6.95%, 5292 images of moderate NPDR at 15.06%, 873 images of severe NPDR at 2.48% and 708 images of Proliferative PDR at 2.02%. Table 1 describes the interpretation of the biomarkers of each class in the Messidor data set. Since the data set contains 25,810 images, 73% of the data set is on natural images, thereby affecting the overall accuracy when evaluating the proposed systems. Considering the vast difference between the normal class and the DR-stage Development classes, we chose 10% of the natural pictures. The natural class contained 2581 pictures (21.69%). The data set contains 11,897 images distributed as follows: 2581 images of normal at 21.69%, 2443 images of mild NPDR at 20.53%, 5292 images of moderate NPDR at 44.48%, 873 images of severe NPDR at 7.43% and 708 images of proliferative PDR at 5.95%. Figure 3a shows samples of the data set representing all classes randomly selected by the system.

### 3.2. Pre-Processing

#### 3.2.1. Improvement of DR Data Set Images

Fundus images contain some artifacts due to the movement of the patient’s eye while taking the images; they also have poor contrast between the microvasculature and their surrounding parts. Noise leads to deterioration in the performance of the systems.

Pre-processing techniques are essential to clear the noise and increase the contrast of the microvasculature. The green channel in the RGB color system provides minute details of the microvasculature and other details of the retina. In the beginning, the average colors for each channel are calculated in RGB. Color constancy is then calculated to re-scale the images. Finally, two overlapping filters are applied: The average filter to remove noise and increase the contrast of the microvasculature and the Laplacian filter to reveal the edges of the microvasculature [26].

First, the average filter is set to a 4 × 4 pixel. The average of the 15 pixels in the kernel is then calculated and used to replace the target pixel. This process is repeated for all pixels in the image, as shown in Equation (1). The equation works by first calculating the average of the 15 pixels in the kernel. This average is then used to replace the target pixel in the output image.

(1) $$S\left(x\right)=\frac{1}{L}\overset{L-1}{\underset{i=0}{\sum }}z\left(x-i\right)\;$$

where $S\left(x\right)$ is the output, $z\left(x-i\right)$ is the previous input and *L* is *L* number pixels of the filter.

Secondly, the Laplacian filter enhances the images by revealing the edges of the microvasculature and black spots. Equation (2) shows how the filtering mechanism works on the pixels of the retinal fundus image.

(2) $$\nabla^{2}f\left(x,y\right)=\frac{\partial^{2}f}{\partial x^{2}}+\frac{\partial^{2}f}{\partial y^{2}}$$

In this equation, the Laplacian of *f* which is a second-order differential operator, is denoted by $\nabla^{2}f$.

Where $\nabla^{2}f$ is the second-order differential equation; and *x*, *y* are the location of pixels in the matrix.

Finally, the two enhanced images are merged by means of the average filter and Laplacian to show the microvasculature more clearly and obtain the improved fundus images as in Equation (3).

(3) $$Final\;enhanced=S\left(x\right)-\nabla^{2}f\left(x,y\right)$$

Figure 3b shows a set of fundus images after the enhancement process. The same images in Figure 3a are displayed in Figure 3b after enhancement.

#### 3.2.2. Data Augmentation Method

The retinal fundus image data set comprises five classes with imbalanced distribution. The moderate class constitutes 44.48% of the data set, while the proliferative class represents 5.95%. This class imbalance could lead to accuracy favoring the majority class, which is a common challenge. Furthermore, preventing overfitting in deep learning models necessitates a substantial data set. To address these issues, a data augmentation technique was employed, which artificially augments images [27]. This technique involves applying operations like rotation, flipping, and shifting to images within the same data set, effectively increasing the size of each class by varying amounts. This process rectifies the imbalance issue and yields a balanced data set. Specifically, the normal and mild classes were augmented by three images per image, the moderate class by one image per image, the severe class by 11 images per image, and the proliferative class by 13 images per image. Consequently, a balanced data set was achieved as Table 2.

Given CNN models’ requirement for extensive training data, the data augmentation process significantly enhances the models’ performance and improves the efficiency of diabetic retinopathy diagnosis.

### 3.3. Hybrid Techniques

Modern techniques consist of CNN and SVM algorithms. The first block is the GoogLeNet and ResNet-18 models that extract features. The dimensions of the features are reduced by the PCA algorithm [28]. The SVM receives features and classifies them with high accuracy and efficiency.

The proposed method has several advantages, which are promising results and low computational cost compared with applying pre-trained CNN models. Hybrid technology requires low-cost computational resources, whereas CNN models require high-cost computing resources.

#### 3.3.1. Deep Feature Extraction

The importance of CNN models lies in the fact that they have many successive layers representing their core. Each layer in CNN has a specific task, and performance is integrated between it and previous and later layers. CNN layers extract features and train the model to classify new test samples. CNN models have the advantage of extracting high-resolution features from many levels in various layers [29]. Each layer extracts a specific type of features; for example, the layer for the color features, the layer for the geometric features, the layer for extracting the texture and shape features, and so on.

ResNet-18 has 18 deep layers distributed into five convolutional, one ReLU, and one average pooling, fully connected and classifies all the input images represented by feature vectors into five appropriate classes. Finally, the softMax activation function has five neurons. ResNet-18 contains layers with different neurons and more than 11.5 million parameters.

The GoogLeNet has 27 layers, including pooling layers. GoogLeNet contains 7 million parameters.

Here, we will briefly discuss the layers used in the proposed system as follows:

Convolutional Layers: CNN contains multiple convolutional layers, each with a specific task. Convolutional layers are the basis of CNN, and their name derives from them. Convolutional layers extract features accurately and efficiently based on parameters that control these layers. These parameters are filter size, zero-padding, and p-step; each parameter has a specific task. The filter size varies from layer to layer and is responsible for the size that the filter *f*(*n*) will convolute around a specific part of the image to be processed *x*(*n*), as shown in Equation (4). The zero padding preserves the image size to be processed according to the original image. The filter moves around the image and moves over the image according to the p-step value [30].

(4) $$z\left(n\right)=\left(x*f\right)\left(n\right)=\overset{\infty }{\underset{-\infty }{\int }}x\left(a\right)f\left(n-a\right)\;da$$

where *f*(*n*) is the filter, *x*(*n*) is the image input, ∗ is convolutional operator and *z*(*n*) is image output.

Pooling layers are essential layers in CNN models. They reduce the dimensions of images and speed up computations. This is important because millions of parameters pass through convolutional layers, which can cause computational problems. Pooling layers solve this problem by reducing the image dimensions using average pooling and max pooling. Average pooling selects a specific part of the image according to the filter size, calculates its average, and then replaces the selected group with a single value (average of all values), as in Equation (5). Max pooling selects a specific part of the image according to the filter size, finds the maximum value, and then replaces the specified group with a single value (maximum value in the given group), as in Equation (6).

(5) $$y\left(i,\;j\right)=\frac{1}{k^{2}}\underset{m,n=1\ldots .l}{\sum }f\left[\left(i-1\right)S+m;\;\left(\;j-1\right)S+n\right]$$

(6) $$y\left(i,\;j\right)=max_{m,n=1\ldots .l}\;f\left[\left(i-1\right)S+m;\;\left(\;j-1\right)S+n\right]$$

where *f* is the filter pixels; *m*, *n* are the location of the matrix; *l* is the size matrix, *l* is the number of pixels selected in each group and *S* is the step filter.

#### 3.3.2. PCA Algorithm

PCA is a statistical method that is used to reduce the dimensionality of data. It works by finding a set of principal components that explain the most variance in the data. In the context of DR, PCA is used to reduce the dimensionality of the features extracted by CNNs. However, the features extracted by CNNs are high-dimensional. This makes it difficult to train CNN models on the features. PCA works by finding a set of principal components that explain the most variance in the data. The principal components are ranked in order of decreasing variance. PCA is used to reduce the dimensionality of the features by selecting the first *k* principal components, where *k* is the desired dimensionality of the features. The first *k* principal components explain the most variance in the data. PCA is used to improve the performance of CNN models on DR data. By reducing the dimensionality of the features, PCA makes it easier for CNN models to learn the relationships between the features and the DR stage. Here are some of the benefits of using PCA to reduce the dimensionality of features extracted by CNNs for DR detection: PCA improves the accuracy of CNN models by reducing the dimensionality of the features. This is because PCA helps to remove noise and irrelevant features from the data. PCA reduces the training time of CNN models by reducing the size of the feature vector. This is because PCA helps to remove redundant features from the data. PCA improves the interpretability of CNN models by reducing the number of features that need to be considered. This is because PCA helps to identify the most important features that are predictive of the DR stage.

#### 3.3.3. SVM Classifier

This section presents features extracted from the CNN models and classified by the SVM algorithm with high accuracy.

The supervised SVM algorithm solves many classification and regression problems. The SVM algorithm selects all data set points in an *n* dimension space (*n* represents the number of features). Each coordinate in a dimensional space represents a specific feature value. The algorithm finds many lines, called hyperplane, and chooses only one representing the max margin between the data points. The algorithm aims to separate features (data points) into classes by hyperplane so it can classify any new data point into its appropriate class with high efficiency. The algorithm selects the support vectors that enable it to choose the best hyperplane. Support vectors are points near or located on the hyperplane that form the max-margin separating the classes. The two types of SVM are linear and nonlinear [31].

**Figure 4:**
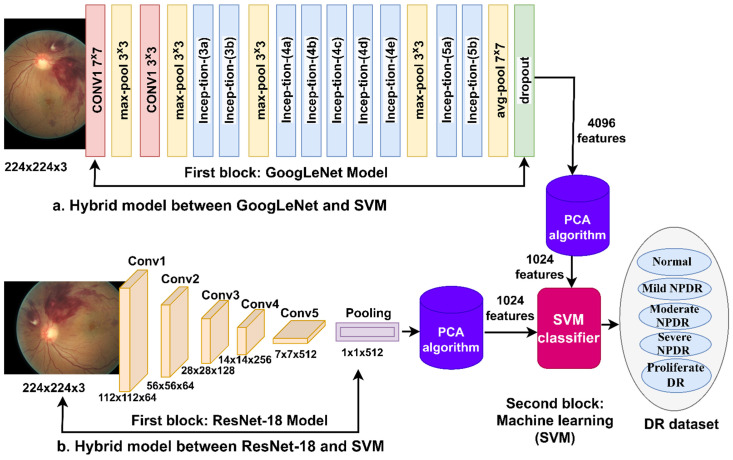
A methodology for fundus imaging diagnostics to diagnose the developmental stages of DR using a hybrid system.

Figure 4 shows a methodology consisting of two blocks: Firstly, GoogLeNet, and ResNet-18 models to extract features and reduce dimensionality using the PCA algorithm; secondly, the SVM for classification [32].

### 3.4. Features Combined with CNN and Handcrafted Features

This section discusses a hybrid method for features extracted from CNN models and handcrafted features. All the features are combined and fed to the FFNN algorithm. The proposed method is characterized by novelty, high accuracy of diagnosis, low computational cost, and need for computer specifications of medium performance and price. This method gives a high representation of the data of each image in the DR data set, and thus promising results are achieved for diagnosing each image with its own class (type of DR intensity).

Handcrafted features such as color, shape, texture, and geometry are important features to represent each image. Thus, combining features of CNN models with handcrafted features yields highly representative feature vectors for each image.

The methodology is as follows: All images are enhanced through average and Laplacian filters. The enhanced fundus images are fed to GoogLeNet and ResNet-18 models to extract features and store them in feature vectors. The feature vectors produced by the CNN models consist of 4096 features for each fundus image [33]. Due to the high feature dimensions, the PCA is applied to reduce the feature to 1024 features so the feature matrix becomes 11,897 × 1024.

The essential features of texture and color are extracted by traditional algorithms Fuzzy color histogram (FCH), Gray Level Co-occurrence Matrix (GLCM), and Local Binary Pattern (LBP) and merged into one feature vector [34]. The FCH algorithm extracts 16 color features for each fundus image, the GLCM algorithm extracts 13 color features and the LBP algorithm extracts 203 features that describe the binary texture. All the features are combined so the size of each feature bus is 232 features, and the feature matrix size is 11,897 × 232.

The FCH method is selected because the microvasculature responds to the RGB color channels, especially the green color channel, which provides fine details of the microvasculature [35]. FCH is a powerful algorithm for extracting the color features based on the fuzzy of each color channel. The GLCM algorithm measures each central pixel (target) of its neighbors based on the distance and angle of the central pixel of its neighbors. GLCM measures spatial and texture relationships. Pixels whose values are close to each other have smooth textures, while pixels whose values are different have rough textures [36]. The LBP has the ability to efficiently extract the texture features of surfaces. Each central pixel in an image is replaced by 24 adjacent pixels through a set operator to 5 × 5 pixels [37].

The features extracted by GoogLeNet are fused with the handcrafted features into feature vectors so that each image has 1256 features. Sixthly, the FFNN classifier is fed with a feature matrix of a size of 11,897 × 1256 to precisely classify it.

The features extracted by ResNet-18 are combined with the features collected by the traditional algorithms (FCH, GLCM, and LBP) into feature vectors so that each image has 1256 features. The FFNN classifier is fed with a feature matrix of a size of 11,897 × 1256 to precisely classify it.

**Figure 5:**
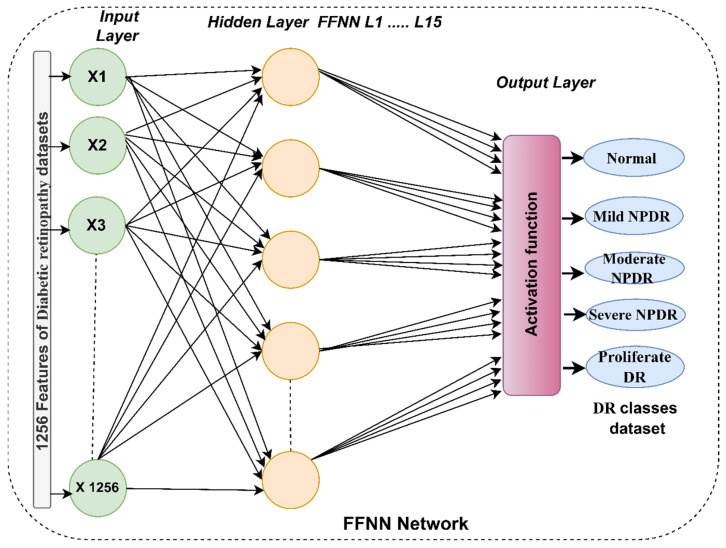
The basic structure of the FFNN network for classifying DR.

The FFNN algorithm is a powerful tool for classifying and predicting medical images. It was used to diagnose fundus images for the detection of DR. The network had 1256 input units, 15 hidden layers, and five output neurons. Each image was classified into its appropriate DR class as shown in Figure 5. The value of each neuron is calculated by calculating the value of the neuron in the previous layer with the weight associated with it. The algorithm works iteratively, updating the weights in each iteration, to calculate the least error of the actual and expected values.

**Figure 6:**
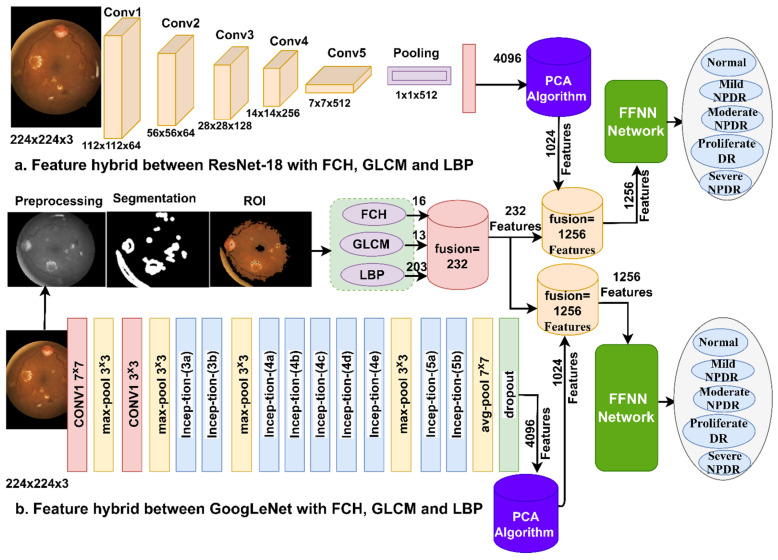
Methodology of hybrid feature extraction and diagnosis by FFNN.

Figure 6 illustrates the methodology of the proposed method, which shows the integration of the features of GoogLeNet and ResNet-18 reduced by the PCA, then combined with handcrafted features. Finally, all the features are provided to the FFNN classifier.

## 4. Experimental Result

### 4.1. Evaluation Metrics

This study used two methods to diagnose DR data sets. The first method used a hybrid technology of CNN with SVM. The second method used hybrid features extracted by CNN (GoogLeNet and ResNet-18) and handcrafted features. All the proposed systems produced a confusion matrix, a table that shows the performance of a classification model. The confusion matrix contains all the data set samples during the testing phase. The performance of the systems was evaluated using Equations (7)–(11), where the required data for the equations were obtained from the confusion matrix [38].

(7) $$\mathrm{Accuracy}=\frac{\mathrm{TN}+\mathrm{TP}}{\mathrm{TN}+\mathrm{TP}+\mathrm{FN}+\mathrm{FP}}*100\%$$

(8) $$\mathrm{Precision}=\frac{\mathrm{TP}}{\mathrm{TP}+\mathrm{FP}}*100\%$$

(9) $$\mathrm{Sensitivity}=\frac{\mathrm{TP}}{\mathrm{TP}+\mathrm{FN}}*100\%$$

(10) $$\mathrm{Specificity}=\frac{\mathrm{TN}}{\mathrm{TN}+\mathrm{FP}}*100\%$$

(11) $$\mathrm{AUC}\;=\frac{\mathrm{True}\;\mathrm{Positive}\;\mathrm{Rate}}{\mathrm{False}\;\mathrm{Positive}\;\mathrm{Rate}}=\frac{\mathrm{Sensitivity}}{\mathrm{Specificity}}*100\%$$

where TP is a DR retinal fundus sample correctly classified as a DR. TN is normal retinal fundus images correctly classified as non-DR. FP is a normal retinal fundus sample classified as DR. FN is a DR retinal fundus image classified as non-DR.

### 4.2. Splitting Data Set

This work aims to diagnose images for the detection of DR development stages by various hybrid methods between deep learning and automatic models and techniques for diagnosing DR based on extracting a mixture of features consisting of color and texture. The data set contains 11,897 images divided into five classes: A class of normal fundus images and four classes representing the stages of DR development. The data set was divided into training and validation by 80% and testing by 20%. Table 3 describes all five classes’ data set distribution. All proposed methods were applied to a PC device with Intel^®^ i5 6th generation processor with specifications of RAM 12 GB and GPU 4 GB. The systems were implemented on MATLAB 2018b environment.

Table 4 describes the training time of the fundus image data set for diabetic retinopathy in all the proposed systems.

### 4.3. Results of CNN Models and Hybrid Techniques

This section discusses the results obtained by pre-trained GoogLeNet and Res-Net-18 models and a hybrid system of CNN and the SVM. Table 5 shows the performance results of pre-trained GoogLeNet and Res-Net-18 for image diagnostics for the detection of DR [39]. The two models achieved results in diagnosing the data set for the detection of DR developmental stages. The GoogLeNet attained 92.56% accuracy, 92.6% precision, 91.8% sensitivity, 98.2% specificity, and 97.42% AUC. In comparison, ResNet-18 obtained 91.47% accuracy, 91.38% precision, 90.2% sensitivity, 97.8% specificity, and 96.58% AUC.

Table 5 shows the implementation results of GoogLeNet + SVM and ResNet-18 + SVM systems to diagnose retinal images for detection of DR. Goog-LeNet + SVM network attained 98.8% accuracy, 97.6% precision, 97.8% sensitivity, 100% specificity, and 98.92% AUC. In comparison, the ResNet-18 + SVM network obtained 98.9% accuracy, 98.8% precision, 98.2% sensitivity, 100% specificity, and 99.21% AUC.

As for the hybrid systems of CNN and the KNN, GoogLeNet, and Res-Net-18 models extract features and store them in feature vectors. The system reduces the feature dimensions by the PCA algorithm.

**Figure 7:**
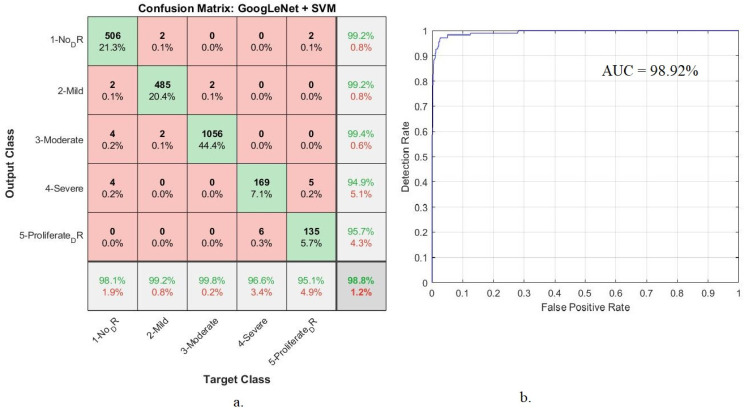
Hybrid technique performance results between GoogLeNet and SVM (**a**) confusion matrix (**b**) AUC.

Hybrid systems have attained good results in the detection of DR stages evolution, as GoogLeNet + SVM attained 98.8% accuracy and 98.92% AUC. The accuracy at each class: The network reached an accuracy of 98.1% for normal, 99.2% for mild, 99.8% for moderate, 96.6% for severe, and 95.1% for proliferative (Figure 7).

**Figure 8:**
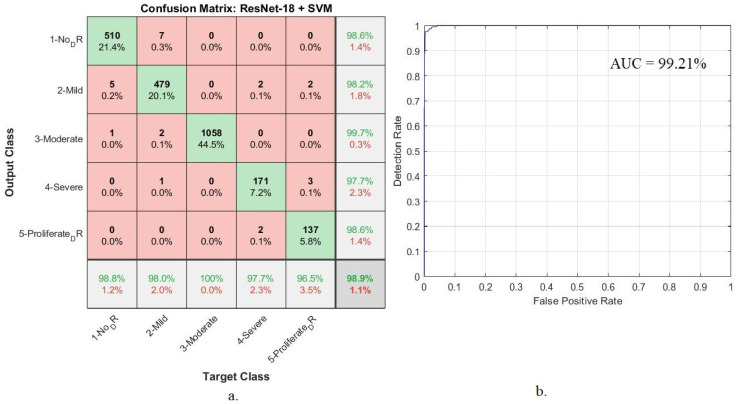
Hybrid technique performance results between ResNet-18 and SVM (**a**) confusion matrix (**b**) AUC.

Also, the ResNet-18 + SVM attained 98.9% accuracy and 99.21% AUC. The accuracy at each class: The network reached an accuracy of 98.8% for normal, 98% for mild, 100% for moderate, 97.7% for severe, and 96.5% for proliferative (Figure 8).

### 4.4. Results of Hybrid Features of CNN and Handcrafted Features

This section shows the evaluation performance of methods by using FFNN with hybrid features of CNN and handcrafted features to diagnose retinal fundus images for detection of DR [40]. The hybrid feature vectors are then fed to the FFNN classifier to achieve superior results. The implementation of the FFNN classifier was adjusted via trial and error at the best performance. This section reviews various FFNN performance assessment tools for diagnosing retinopathy data sets.

The confusion matrix is the gold standard for evaluating performance systems. In this study, the performance of FFNN was evaluated based on features of CNN models and handcrafted.

Table 6 shows the implementation of the FFNN classifier based on the hybrid features of fundus image diagnostics for the detection of DR developmental stages. The network reached promising results due to the hybrid and diverse features of several algorithms.

This section discusses two models of GoogLeNet-handcrafted-FFNN and ResNet-18-handcrafted-FFNN performance experiments where promising results were achieved in two experiments. First, GoogLeNet-handcrafted-FFNN attained 99.6% accuracy, 99.4% precision, 99.2% sensitivity, 100% specificity, and 99.78% AUC. By contrast, the ResNet-18-handcrafted-FFNN attained 99.7% accuracy, 99.6% precision, 99.6% sensitivity, 100% specificity, and 99.86% AUC.

**Figure 9:**
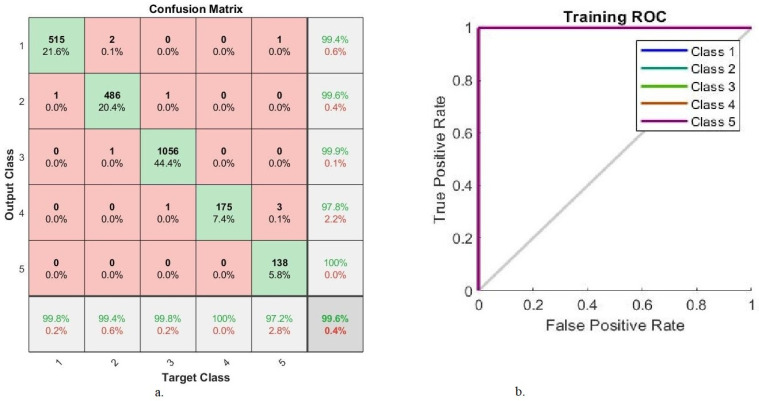
GoogLeNet-handcrafted-FFNN performance results (**a**) confusion matrix (**b**) AUC.

Figure 9 describes the AUC and confusion matrix generated by GoogLeNet-handcrafted-FFNN, which attained an overall 99.6% accuracy and 99.78% AUC. It also attained superior accuracy for diagnosing each stage of DR development, where the network attained an accuracy of 99.8%, 99.4%, 99.8%, 100%, and 97.2% for the diagnoses of class 1 (normal), class 2 (mild), class 3 (moderate), class 4 (severe), and class 5 (proliferative) stages, respectively.

**Figure 10:**
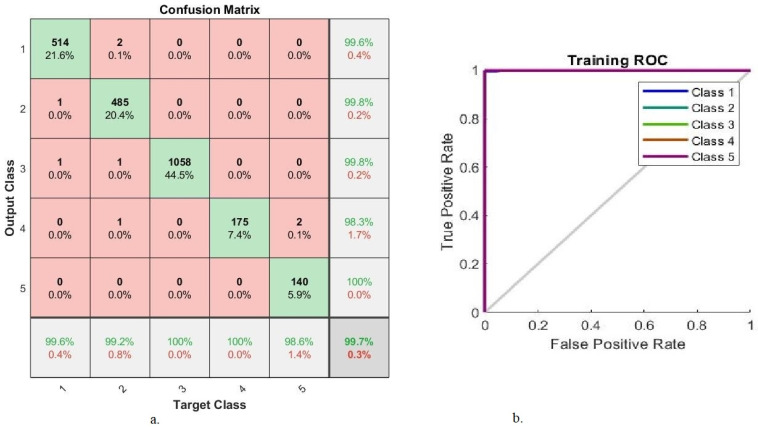
ResNet-18-handcrafted-FFNN performance results (**a**) confusion matrix (**b**) AUC.

Figure 10 describes the AUC and confusion matrix generated by ResNet-18-handcrafted-FFNN, which attained an overall 99.7% accuracy and 99.86% AUC of 99.86%. It also achieved superior accuracy in diagnosing each stage of DR development. The system attained an accuracy of 99.6%, 99.2%, 100%, 100%, and 98.6% for the diagnoses of class 1 (normal), class 2 (mild), class 3 (moderate), class 4 (severe) and class 5 (proliferative) stages, respectively.

In Figure 10b, each color represents one class in the data set.

## 5. Discussing the Performance of the Systems

In this study, two methods were discussed; each technique has two systems (two experiments), and each experiment has a different method and materials. The study aimed to accurately diagnose the retinal fundus for detecting DR developmental stages. Since fundus images contain noise and low micro-vascular contrast, all images were enhanced. The imbalance of the data set and the lack of the data set led to poor performance of the system and the accuracy bias to the majority class, so the data augmentation method was used to balance the data set by increasing the number of images for each class by an amount different from the other class [41].

The reason for using GoogLeNet and ResNet-18 models is to produce more convenient features when combined with handcrafted features.

The first method is a hybrid system of CNN (GoogLeNet and ResNet-18) and SVM. GoogLeNet + SVM and ResNet-18 + SVM, reaching an accuracy of 98.8% and 98.9%, respectively.

The second proposed method uses FFNN based on features CNN with handcrafted features. The features were extracted from CNN models and then reduced by the PCA. Finally, all the resulting features are combined using first, GoogLeNet-handcrafted-FFNN; Second, ResNet-18-handcrafted-FFNN. GoogLeNet-handcrafted-FFNN attained an accuracy of 99.6%. In comparison, ResNet-18-handcrafted-FFNN attained an accuracy of 99.7%.

Table 7 describes the results of all methods for fundus image diagnostics for the detection of DR developmental stages. The table shows the overall accuracy of each technique and the accuracy of the diagnosis at the level of each stage of DR. For normal fundus images, the GoogLeNet-handcrafted-FFNN model attained 99.8%. GoogLeNet-handcrafted-FFNN reached 99.4% for the diagnosis of the mild stage. For the moderate stage, ResNet-18 + SVM and ResNet-18-handcrafted-FFNN attained 100%. FFNN reached 100% accuracy in diagnosing the severe stage based on the hybrid features. Finally, the ResNet-18-handcrafted-FFNN attained 98.6% accuracy for proliferative stage diagnosis.

**Figure 11:**
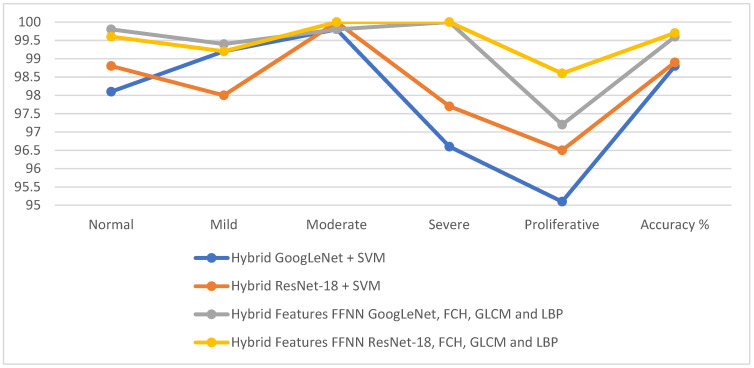
Display of the performance of the proposed methods in this study for diagnosing the developmental stages of DR.

Figure 11 presents the results of evaluating the proposed methods in this study to diagnose fundus images to detect stages of DR.

Table 8 has been added to compare the performance of all the methods proposed in this study. Where it is noted that the Res-Net-18-handcrafted-FFNN model is superior to the rest of the proposed systems in all measures of accuracy, precision, sensitivity, and specificity.

Table 9 shows the results of previous studies related to diagnosing a fundus image data set for the detection of DR and compares them with the results of the proposed systems. Our proposed system is better than previous approaches in all measures. The previous systems attained an accuracy of between 65.2% and 97%, while the proposed system attained 99.7% accuracy. The previous systems attained a sensitivity between 64.2% and 98.48%, while the proposed system attained 99.6% sensitivity. The previous systems attained a specificity of between 66.2% and 98%, while the proposed system attained 100% specificity. The previous systems attained an AUC of between 92.72% and 97.3%, while the proposed system attained 99.86% AUC.

## 6. Conclusions

The rapid spread of diabetes and the swelling number of people with diabetes worldwide require many highly trained ophthalmologists. The development of DR has many stages, ranging from mild to moderate to severe PDR. If early diagnosis of the initial stages is not performed, then it will lead to the stage of PDR that causes severe vision impairment and blindness. The treatment of DR in its early stages requires highly qualified experts and takes a long time. Systems using artificial intelligence have been developed to overcome the shortcomings of manual diagnosis. In this study, two proposed methods were developed, each with two experiments. The first method is to use hybrid systems of CNN and SVM. This method reached promising results in fundus imaging diagnostics to detect the stages of DR. The second proposed method is FFNN with features of CNN and handcrafted. The ResNet-18-handcrafted-FFNN achieved an excellent performance, with 99.7% accuracy, 99.6% precision, 99.6% sensitivity, 100% specificity, and 99.86% AUC.

The limitations encountered include an unbalanced data set and a lack of sufficient images to train the data set. These limitations can be overcome by applying data augmentation.

Future works on the features of many CNN models will be combined and classified by ANN, FFNN, random forest, decision trees, and AdaBoost classifiers.

## Figures and Tables

**Table 1:** Classification of DR with appearance of biomarker.

Stages of DR	Lesion Detection	No of Images
Normal	It is normal and no abnormalities were noticed	25,810
Mild NPDR	The appearance of aneurysms lightly	2443
Moderate NPDR	Appearance of microvascular aneurysm with an amount of more than Mild NPDR and less than Severe NPDR	5292
severe NPDR	Spotted macular bleeding in the four quadrants Microvascular abnormalities in at least one quadrant The appearance of blood vessel protrusion in one of the quadrants	873
PDR	Appearance of pre-retinal hemorrhage—Appearance of Neovascularization	708

**Table 2:** Augmentation method of balancing the DR data set through the training stage.

Phase	Training Phase				
Class Name	Normal	Mild	Moderate	Severe	Proliferative
Before augmentation	1652	1563	3387	558	453
After augmentation	**6608**	**6252**	**6774**	**6696**	**6342**

**Table 3:** Splitting of retinal fundus images during all stages for all classes.

Phase	Training and Validation 80%		Testing 20%
Classes	Training (80%)	Validation (20%)	
Normal	1652	413	516
Mild	1563	391	489
Moderate	3387	847	1058
Severe	558	140	175
Proliferative	453	113	142

**Table 4:** Training time of the proposed systems.

Techniques	Extracting Features Methods	Training Time	Testing Time
CNN	GoogLeNet	320 min 54 s	13 min 49 s
	ResNet-18	280 min 39 s	11 min 8 s
Hybrid	GoogLeNet + SVM	5 min 26 s	1 min 52 s
	ResNet-18 + SVM	4 min 9 s	1 min 14 s
FFNN	GoogLeNet and handcrafted	11 min 18 s	2 min 42 s
	ResNet-18 and handcrafted	9 min 31 s	2 min 17 s

**Table 5:** CNN and Hybrid models performance results for detection of DR stages.

Methods	CNN Models		Hybrid Models	
Measure	GoogLeNet	ResNet-18	GoogLeNet + SVM	ResNet-18 + SVM
Accuracy %	92.56	91.47	98.8	98.9
Precision %	92.6	91.38	97.6	98.8
Sensitivity %	91.8	90.2	97.8	98.2
Specificity %	98.2	97.8	100	100
AUC %	97.42	96.58	98.92	99.21

**Table 6:** Performance of FFNN based on hybrid features to detect DR stages.

Hybrid Features	GoogLeNet-Handcrafted-FFNN	ResNet-18-Handcrafted-FFNN
Accuracy %	99.6	99.7
Precision %	99.4	99.6
Sensitivity %	99.2	99.6
Specificity %	100	100
AUC %	99.78	99.86

**Table 7:** Performance of the proposed methods in this study to diagnose fundus images to reveal the developmental stages of DR.

Diseases			Normal	Mild	Moderate	Severe	Proliferative	Accuracy %
Hybrid		GoogLeNet + SVM	98.1	99.2	99.8	96.6	95.1	98.8
		ResNet-18 + SVM	98.8	98	100	97.7	96.5	98.9
Hybrid Features	FFNN	GoogLeNet, FCH, GLCM and LBP	99.8	99.4	99.8	100	97.2	99.6
		ResNet-18, FCH, GLCM and LBP	99.6	99.2	100	100	98.6	99.7

**Table 8:** Performance of all proposed methods.

Methods	CNN Models		Hybrid Models		CNN-Handcrafted-FFNN	
Measure	GoogLeNet	ResNet-18	GoogLeNet + SVM	ResNet-18 + SVM	GoogLeNet-Handcrafted-FFNN	ResNet-18-Handcrafted-FFNN
Accuracy %	92.56	91.47	98.8	98.9	99.6	99.7
Precision %	92.6	91.38	97.6	98.8	99.4	99.6
Sensitivity %	91.8	90.2	97.8	98.2	99.2	99.6
Specificity %	98.2	97.8	100	100	100	100
AUC %	97.42	96.58	98.92	99.21	99.78	99.86

**Table 9:** Comparison of the performance results of proposed systems with relevant previous studies.

Previous Studies	Accuracy %	Sensitivity %	Specificity %	AUC %
Liu et al. [12]	85.44	98.48	71.82	-
Qummar et al. [13]	65.2	64.2	66.2	-
Gao et al. [14]	85.5	94	93.01	-
Wan et al. [16]	93.36	77.66	93.45	92.72
Romany et al. [18]	95.26	96	93	-
Shanthi et al. [19]	96.6	-	-	-
Martinez et al. [21]	95.5	98.3	94.5	97.3
Hemanth et al. [22]	97	94	98	-
**Proposed model**	**99.7**	**99.6**	**100**	**99.86**

## Data Availability

The data that supports the results of the evaluation of the performance of the proposed methods in this study was collected through a data set available online at the link: https://www.kaggle.com/competitions/diabetic-retinopathy-detection/data (18 October 2022).
